# Methylglyoxal Scavengers Attenuate Angiogenesis Dysfunction Induced by Methylglyoxal and Oxygen-Glucose Deprivation

**DOI:** 10.1155/2022/8854457

**Published:** 2022-01-05

**Authors:** Wei Chen, Wenhui Huang, Yu Yang, Keshen Li

**Affiliations:** ^1^Clinical Neuroscience Institute, The First Affiliated Hospital of Jinan University, 510632 Guangzhou, China; ^*^2^*^ Department of Neurology and Stroke Center, The First Affiliated Hospital of Jinan University, 510632 Guangzhou, China

## Abstract

Cerebral endothelial cells play an essential role in brain angiogenesis, and their function has been found to be impaired in diabetes. Methylglyoxal (MG) is a highly reactive dicarbonyl metabolite of glucose formed mainly during glycolysis, and its levels can be elevated in hyperglycemic conditions. MG is a potent precursor of AGEs (advanced glycation end-products). In this study, we investigated if MG can induce angiogenesis dysfunction and whether MG scavengers can ameliorate angiogenesis dysfunction induced by MG. Here, we used cultured human brain microvascular endothelial cells (HBMECs) treated with MG and oxygen-glucose deprivation (OGD) to mimic diabetic stroke in vitro. We also used the MG challenged chicken embryo chorioallantoic membrane (CAM) to study angiogenesis in vivo. Interestingly, administration of MG significantly impaired cell proliferation, cell migration, and tube formation and decreased protein expression of angiogenesis-related factors, which was rescued by three different MG scavengers, glyoxalase 1 (GLO1), aminoguanidine (AG), and N-acetyl cysteine (NAC). In cultured CAM, MG exposure significantly reduced angiogenesis and the angiogenesis-related dysfunction could be attenuated by pretreatment with AG or NAC. Treatment of cultured HBMECs with MG plus OGD increased cellular apoptosis significantly, which could be prevented by exposure to GLO1, AG, or NAC. We also noted that administration of MG increased cellular oxidative stress as measured by reactive oxygen species (ROS) generation, enhanced AGE accumulation, and receptor for advanced glycation end-product (RAGE) expression in the cultured HBMECs, which were partially reversed by GLO1, AG, or NAC. Taken together, our findings demonstrated that GLO1, AG, or NAC administration can ameliorate MG-induced angiogenesis dysfunction, and this can be mainly attributed to attenuated ROS production, reduced cellular apoptosis, and increased levels of angiogenic factors. Overall, this study suggested that GLO1, AG, or NAC may be promising candidate compounds for the treatment of angiogenesis dysfunction caused by hyperglycemia in diabetic ischemic stroke.

## 1. Introduction

Ischemic stroke is a leading cause of long-term disability that has been found to seriously endanger human life and health worldwide. Ischemic stroke makes up approximately 87% of all stroke cases and results in heavy burden on families and society. Ischemic stroke is characterized by transient or permanent local reduction of cerebral blood flow because of occlusion of cerebral arteries. Therefore, extensive research has been focused to restore or improve the reduction of regional cerebral blood supply for improving cerebral deficits and promoting poststroke functional recovery [1, 2]. It has been demonstrated that rapid angiogenic response to form new vessels can be quite effective to increase the cerebral blood flow [3, 4], thereby indicating that the angiogenesis may play a key role in neurological recovery after stroke. For example, Lu et al. [5] demonstrated that overexpression of netrin-1 before middle cerebral artery occlusion (MCAO) in mice increased focal angiogenesis, reduced infarct size, and promoted long-term beneficial functional outcome. Angiogenesis refers to the proliferation, migration, and tube formation of endothelial cells to generate new blood vessels in a budding manner in the original blood vessels [6]. The angiogenic response is associated with many key angiogenic factors such as vascular endothelial growth factor (VEGF) and VEGF receptor 2 (VEGFR-2) in the ischemic tissue, and these molecules have been found to not only initiate angiogenesis but also play an important role in the development of angiogenesis [7].

Diabetes is an important risk factor for ischemic stroke, and they often arise together. Accumulating evidence has demonstrated that the neurological dysfunction of ischemic stroke is more serious in patients with diabetes, as compared with those not having diabetes [8, 9]. Furthermore, the mortality of ischemic stroke patients with diabetes is significantly increased compared with that of ischemic stroke patients without diabetes [10, 11]. Diabetes mellitus impairs endothelial cell function, decreases vascularity as well as causes postischemic reparative neovascularization [12], and thus seems to play a critical role in diabetic stroke. Hyperglycemia arises through inactivation of the hypoxia-inducible factor-1a (HIF-1*α*) that can regulate angiogenic factors, including VEGF [13, 14]. MG, a highly reactive dicarbonyl compound, is formed during glucose metabolism, and its blood levels can be significantly elevated in diabetes [15, 16]. Moreover, AGE interaction with their cognate receptors can increase oxidative stress by inducing the formation of ROS that can significantly contribute to the cerebrovascular dysfunction in diabetes [17]. However, there are only few published reports about the possible influence of MG on angiogenesis in the event of an ischemic stroke. The glyoxalase system including glyoxalase-1 (GLO1), glyoxalase-2 (GLO 2), and GSH can catalyze the conversion of MG to D-lactate via the intermediate S-D-lactoylglutathione. In addition, the transgenic mice overexpressing GLO1 in bone marrow cells can reverse defective neovascularization in STZ-induced diabetic mice [18].

The aim of the current study was to use human brain microvascular endothelial cells (HBMECs) to explore if MG impaired cell proliferation, cell migration, and tube formation leading to angiogenesis dysfunction under OGD. In addition, we have used CAM model to observe the angiogenesis response after MG treatment in vivo. We also examined whether two different MG scavengers, aminoguanidine (AG) and N-acetylcysteine (NAC), could significantly restore endothelial function and reverse the defective angiogenesis induced by MG during ischemia.

## 2. Materials and Methods

### 2.1. Reagents and Chemicals

Methylglyoxal, aminoguanidine (AG, Product Number: A7009), and N-acetylcysteine (NAC, Product Number: A0737) were purchased from Sigma (St Louis, MO, USA). The inhibitor of glyoxalase1, S-p-bromobenzylglutathione-cyclopentyl-diester (BBGC), was purchased from Sigma (St Louis, MO, USA). Rabbit anti-AGE polyclonal antibody was purchased from Abcam (1 : 1000, Cambridge, MA, USA). Anti-VEGFA (1 : 500), anti-VEGFR1 (1 : 500) polyclonal antibodies, and Human VEGF ELISA Kit were purchased from Novus Biologicals (Centennial, CO, USA). Anti-*β*-actin (1 : 1000), anti-p-VEGFR2 (1 : 1000), and anti-Notch1 (1 : 1000) polyclonal antibodies were purchased from Cell Signaling Technology (Beverly, MA, USA). Anti-RAGE (1 : 1000) and anti-HIF-1*α* (1 : 1000) polyclonal antibodies were purchased from Santa Cruz Biotechnology (Bolivia, Northern California, USA). Immunofluorescence staining was performed on CAM using monoclonal antibodies caveolin 1 (1 : 200, Santa Cruz, TX, USA). All the western blotting secondary antibodies were purchased from Beyotime Biotechnology (1 : 1000, Shanghai, China). Reactive oxygen species (ROS) assay kit and Cell Counting Kit-8 (CCK-8) were purchased from Beyotime Biotechnology (Shanghai, China). Matrigel was purchased from BD (Centennial, CO, USA). Annexin V-APC/7-AAD Apoptosis Detection Kit was obtained from Biotechnology (Hubei, Wuhan, China). Total RNA was isolated from using a Trizol kit (Invitrogen, CA, USA). First-strand cDNA and SYBR Green qPCR assays were performed using a PrimeScript RT reagent kit (Takara, Shiga-ken, Japan).

### 2.2. Cell Culture and Treatment Protocols

Human brain microvascular endothelial cells (HBMECs) were a kind gift from Prof. Zhijun Zeng. The cells were plated in T25 culture flask and grown in complete growth media Dulbecco's modified Eagle's medium (DMEM) supplemented with 10% Fetal Bovine Serum (FBS), 100 U/mL penicillin, and 100 mg/mL streptomycin. The cultures were maintained at 37°C in a humidified atmosphere at 5% CO_2_. 24 hours after the cells were inoculated, it was observed that 80% of the cells were adherent, which could be treated with drugs and infected with viruses. HBMECs were allowed to grow 80% confluency before treatment with MG for 24 h in the presence of the selective inhibitor of glyoxalase-1, S-p-bromobenzylglutathione-cyclopentyl-diester (BBGC) (10 mmol/L), as previously described [19].

### 2.3. Vectors and Lentivirus Infection

Lentiviral GLO1 (LV- GLO I) and empty lentiviral vector (LV–NC) were constructed by Genechem Co. (Shanghai, China) and transfected to the HBMEC according to the manufacturer's instructions. The efficiency of transfection was detected by a fluorescence microscope and qRT-PCR.

### 2.4. Oxygen-Glucose Deprivation and Reperfusion

To mimic ischemia/reperfusion conditions in vitro, OGD treatment was performed on HBMECs according to a previous report with minor modifications [20]. In brief, the glucose-free DMEM culture medium was first saturated in a hypoxic incubator at 37°C to zero oxygen for 30 min. The cells were washed with PBS twice and incubated in the prepared hypoxia medium for 4 h in the hypoxia chamber (5% CO_2_, 1% O_2_, and 94% N_2_). After 4 h of OGD exposure, the HBMECs were maintained in normal culture conditions for reoxygenation for 20 h according to the experimental design. The control cells were not exposed to OGD.

### 2.5. Assessment of Cell Viability and Apoptosis Assay

The extent of HBMEC viability was measured by the commercially available enhanced Cell Counting Kit-8 (CCK-8) according to the manufacturer's instructions. Briefly, exponentially growing cultured HBMECs were plated at a density of 1 × 10^3^ cells per well in 96-well plates overnight and were exposed to various treatments for 24 h. Subsequently, the cells were washed and incubated with CCK-8 solution (10 *μ*L) at 37°C for 1 h. The plate absorbance was measured at 450 nm in a microplate reader (Thermo Fisher Scientific). Percent over control was calculated to measure the cell viability. Annexin V-APC/7-AAD with flow cytometric analysis was used to measure cell apoptosis. In this experiment, 5 × 10^5^ cells in 100 *μ*L 1× Annexin V Binding Buffer were stained with 2.5 *μ*L Annexin V-APC and 2.5 *μ*L 7-AAD at room temperature for 20 min, respectively. Annexin V-APC and 7-AAD were mixed together and were then analyzed by flow cytometry using a BD FACSCalibur flow cytometer (Becton Dickinson, Missouri, TX, USA).

### 2.6. Cell Migration and Tube Formation Assay

The migration ability of HBMEC was examined in a 6-well plate according to the manufacturer's protocol (Corning, NY, USA). Briefly, the cells were suspended in complete culture medium and seeded into the 6-well plate at a concentration of 1 × 10^5^ cells per well for 24 h. Twenty-four hours after GLO transfection, NAC, and AG drug treatment, HBMEC was grown to confluence in a 6-well plate, and a small scratch was made in the monolayer of HBMEC using a P10 pipette tip as described previously. MG (1 mmol/L) was cocultured with HBMEC in the cell migration assay to block cell proliferation. Twenty-four hours later, the extent of wound healing was determined by the distance between the leading edges of the migrating HBMEC. The images of distance was obtained by a computer-assisted microscope and analyzed by ImageJ software (National Institutes of Health, Bethesda). Statistical analyses of data are performed in three independent experiments.

In vitro tube formation assay was performed according to the manufacturer's instructions. Matrigel (200 *μ*L) was coated in a 24-well plate and incubated at 37°C for 30 min to allow for polymerization. HBMEC was resuspended and seeded in a 24-well plate at a density of 5 × 10^5^/well. Subsequently, HBMEC was cocultured with different drug treatments for 24 h. Tube formation capability in each well was examined by a computer-assisted microscope after 4 h and 8 h. Quantification was analyzed by using Image J software. We calculated the degree of total tube length and total number of branches in at least five images from different areas of each sample. Statistical analyses of data are performed in three independent experiments.

### 2.7. Enzyme-Linked Immunosorbent Assay (ELISA)

VEGF secretory protein levels were detected by ELISA (enzyme-linked immunosorbent assay) after treatment with different drugs and OGD/R for 24 h. The cell culture supernatants were collected from HBMECs, and the protein level of VEGF was measured using ELISA kits (Novus Biologicals, Centennial, CO, USA) according to the manufacturer's instructions.

### 2.8. Measurement of ROS Release

ROS production was used to measure the level of oxidative stress. HBMEC was seeded in a 6-well plate at a density of 2 × 10^5^ cells per well and incubated for 24 h at 37°C. Then, HBMECs were treated with MG (1 mmol/L) plus OGD/R in the presence or absence of GLO I NAC or AG, incubated at 37°C for 24 h. The intracellular ROS levels were determined with a DCFH-DA cellular ROS detection assay kit purchased from Beyotime Biological Industry (Shanghai, China). After adding 10 *μ*M DCFH-DA dissolved in serum-free medium to each well, plates were incubated at 37°C for 20 min protected from light. After incubation for 20 min, each well was washed by phosphate-buffered saline (PBS) to remove residual DCFH-DA. Plates were then read in a fluorescence microscope and analyzed by ImageJ.

### 2.9. Chorioallantoic Membrane Model (CAM) Assay

The fertilized chick eggs were obtained from the Avian Farm of the South China Agriculture University. The fertilized chicken eggs were used for CAM assay to measure angiogenesis in vivo as described previously [21]. The eggs were incubated at 37°C with a relative humidity of 70% in an incubator. After 36 h, a small hole about 1 × 1 mm in size was opened in the shell and different drugs and control vehicle were added into and the CAM. The dosing was done once every 3 days, for a total of 3 times. The window is sealed with liquid paraffin. On day 9, the embryo and its extraembryonic membranes were transferred to a Petri dish and washed by PBS three times. Subsequently, the blood vessel in the CAM model was captured in a stereo microscope and neovascularization density was analyzed by using IPP software.

### 2.10. Hematoxylin/Eosin (HE) Staining of CAM

For hematoxylin/eosin staining, CAM were fixed with phosphate-buffered saline (pH 7.4) in 4% paraformaldehyde, and the samples were then dehydrated and embedded in paraffin wax, followed by sectioning (3 *μ*m thickness). Subsequently, the sections were baked for 30 min at 55°C, hydrated through a graded ethanol series, stained for 10 min with hematoxylin, and then stained for 5 min with eosin. The sections were washed by PBS and then employing graded alcohol to dehydrate and xylene to increase transparency and thereby mounted with neutral gum. The changes in specimens were captured using an optical microscope.

### 2.11. Immunofluorescence Staining

Paraformaldehyde-fixed paraffin sections of chorioallantoic membrane prepared previously were used for immunofluorescence staining. Paraffin sections were dewaxed in xylene and hydrated. After being washed 3 times with PBS, they were then placed in sodium citrate antigen repair solution in a microwave over high heat for 6 min. Subsequently, the slices were incubated with 10% donkey serum with 0.3% Triton X-100 in PBS for 1 h. The sections were thereafter incubated overnight with the following primary antibodies at 4°C: rabbit anti-caveolin1. After washing 3 times with PBS, the slides were further incubated with fluorescence-labeled secondary antibodies at room temperature for 1 h and stained with DAPI for 5 min. Thereafter, the images were captured using a laser scanning confocal microscope (Leica, Heidelberg, Germany).

### 2.12. Western Blotting Analysis

We used the embryonic eggs cultured up to the 9th day; removed the chicken embryos, leaving only the allantoic membrane tissue; and thereafter placed them in a dish containing DEPC water to expand the remaining blood. The tissue was then placed into the EP tube and incubated on ice; thereafter, 100 *μ*L of ready-to-use protein lysis buffer was added per 20 mg. Thereafter, two magnetic beads were added in each tube, placed on an oscillator, and shaken for 70 s at 4°C, 90 Hz, 3 cycles. The tissue was then ground for homogenization and then placed in an ultrasonic disruptor for 10 minutes. Thereafter, it was centrifuged at 12000 rpm, for 5 min, at 4°C. After centrifugation, the supernatant was collected, transferred in a new EP tube, and stored at -80C. The total protein was extracted using a cell lysis buffer for western blot and IP (Beyotime Biotechnology, Shanghai, China) and a protease inhibitor cocktail set I-Calbiochem (Merck Millipore, Billerica, MA, USA). The amount of protein in supernatants was determined by using a Beyotime protein assay kit (Beyotime Biotechnology, Shanghai, China). The proteins were separated by 8-12% SDS-PAGE and transferred to 0.4 mm PVDF membranes (EMD Millipore, Billerica, MA, USA). After 1 h blocking in 5% nonfat milk, the membranes were incubated with primary antibody at 4°C overnight. The membranes were incubated with specific horseradish peroxidase–conjugated secondary antibody at room temperature for 1 h and developed with enhanced chemiluminescence reagent (Merck Millipore). The protein band analysis and quantification were performed using ImageJ software.

### 2.13. Quantitative Real-Time PCR (qRT-PCR)

RNA extraction and quantitative real-time PCR (qRT-PCR) were performed to evaluate the expression of mRNAs. Total RNA was isolated from the HBMEC using Trizol reagent. Total RNA was used to synthesize the mRNA first-stranded cDNA according to the manufacturer's protocol. Quantitative real-time PCR was performed with CFX96™ Real-Time System (Bio-Rad, Hercules, CA, USA) using an initial step at 95°C for 15 s for denaturation and 60°C for 60 s for annealing followed by 40 cycles according to the manufacturer's recommendations, with *β*-actin used as an internal control. Data were analyzed using the comparative Ct method (2^-*ΔΔ*Ct^) and normalized to *β*-actin. The sequences of PCR primers (5′-3′) used were GCTTCTTCCTGCGCATCAACC and TCCTCCTTCATAGCCAGAAAGC for FGF-2; ATGAACTTTCTGCTCACTTG and TCACCGTCTCGGTTTTTCAC for VEGFA; and AGGCAGTTGGAATTGGGTCT and CAGATATATTGCATGAGGAACTGC for HIF-1*α*.

### 2.14. Statistical Analysis

The data has been shown as means ± standard deviation (SD). Statistical analysis was performed using SPSS 21.0 (IBM, Armonk, NY, USA). For all experiments, the number of independent experiments (*n*) has been indicated. The one-way analysis of variance (ANOVA) of Fisher's least significant difference test or the unpaired 2-tailed Student's *t*-test was used for statistical analysis. The difference was considered statistically significant when *p* values were <0.05.

## 3. Results

### 3.1. Cytoprotective Effects of GLO1, NAC, and AG in HBMEC after MG and OGD Exposure

**Figure 1:**
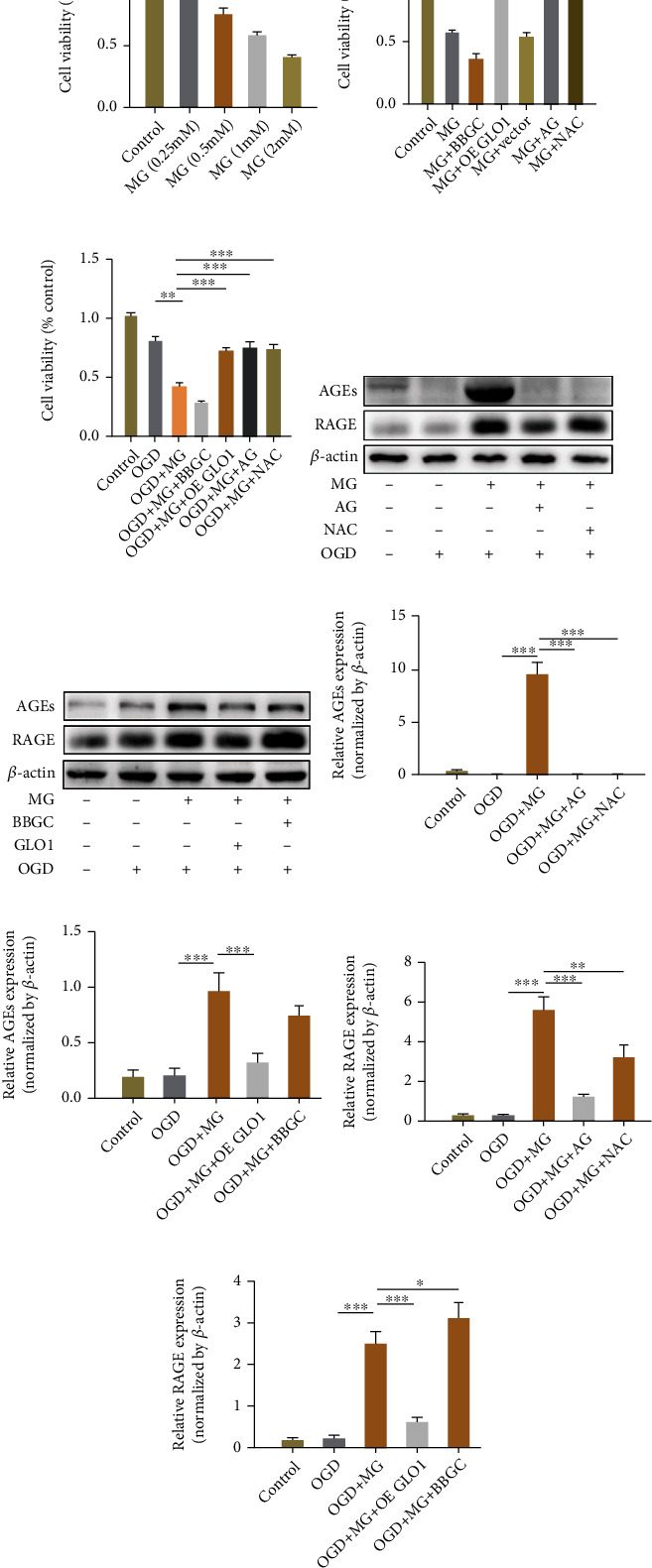
Cytoprotective effects of GLO1, NAC, and AG in HBMEC following MG and OGD exposure. (a–c) HBMECs were exposed to different drugs for 24 hours, and the cell viability was measured by CCK-8 assay. (d, e) The expression of various proteins was determined by western blotting. (f–i) Quantitative analysis of protein expression levels. All data are the means ± SD (*n* = 3), ^∗^*p* < 0.05, ^∗∗^*p* < 0.01, and ^∗∗∗^*p* < 0.001 vs. the MG group.

To determine the cytoprotective effect of GLO1, NAC, and AG in HBMEC following MG exposure, we performed the CCK-8 assay to study the effects on cell viability and cytotoxicity. The virus infection effect has been supplemented in the Supporting Information. Firstly, the cells were treated with 0.25, 0.5, 1, and 2 mmol/L MG for 24 h and cell viability was determined by CCK-8 assay (Figure 1(a)). Cell viability was found to decrease in a dose-dependent manner to 93.60 ± 4.10%, 73.70 ± 6.30%, 57.00 ± 3.80%, and 39.80 ± 2.50%, respectively, after 24 h MG incubation. Secondly, GLO1 (MOI = 100), NAC (2 mmol/L), and AG (2 mmol/L) pretreatment significantly attenuated 1 mmol/L MG-induced stress (*p* < 0.001) as compared to the MG exposure alone (Figure 1(b)). Thirdly, we performed CCK-8 assay under 24 h MG and 4 h OGD exposure to mimic diabetes-enhanced ischemic HBMEC injury. As shown in Figure 1(c), the viability was significantly decreased in cells treated with MG plus OGD (41.90 ± 3.20%) as compared with the OGD-treated group (88.00 ± 5.20%). Upon GLO 1 and MG scavenger and NAC as well as AG pretreatment, the cell viability was increased to 72.50 ± 3.00%, 73.60 ± 4.30%, and 74.90 ± 5.20%, respectively. MG is a glycolytic metabolism byproduct that can be accumulated in diabetic patients and a precursor of AGEs. It has been reported that AGEs can interact with specific receptor RAGE and play a key role in MG-induced stress. In our study, we observed that GLO1, NAC, and AG reversed MG plus OGD-induced cytotoxicity in HBMEC by significantly decreasing AGE accumulation and RAGE expression (Figures 1(d)–1(i)).

### 3.2. GLO1, NAC, or AG Protected HBMEC from Apoptosis

**Figure 2:**
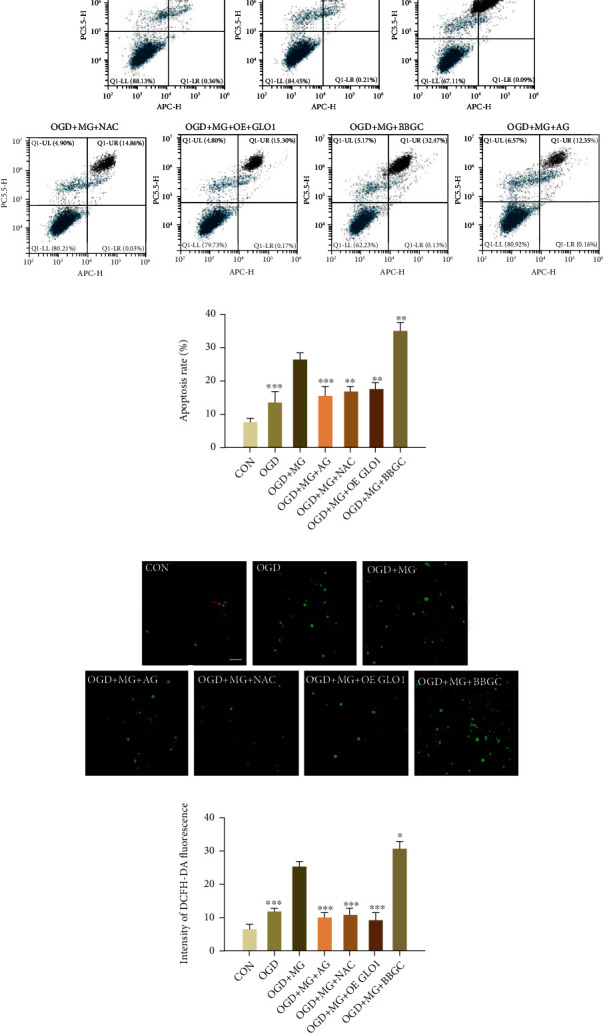
GLO1, NAC, or AG protected HBMEC from apoptosis. (a) Annexin V-APC/7-AAD flow cytometry assay was used to detect apoptosis in different groups. (b) The percentages of apoptotic cells were assessed by flow cytometry. (c) ROS production was determined by the fluorescence intensity of DCFH-DA. (d) The bar graph shows relative increase in fluorescence which indicates ROS levels. All data are means ± SD (*n* = 3), ^∗^*p* < 0.05, ^∗∗^*p* < 0.01, and ^∗∗∗^*p* < 0.001 vs. the MG group.

To determine whether cell cytotoxicity induced by MG can also contribute to apoptosis induction, we used an Annexin V-APC/7-AAD doubled-labeled flow cytometry analysis for verification in HBMECs. The results showed that the proportion of apoptosis was significantly increased in OGD plus MG-treated cells as compared with OGD-treated cells, whereas GLO 1, NAC, or AG pretreatment significantly decreased cellular apoptosis from 26.35 ± 1.88% to 17.50 ± 1.97%, 16.69 ± 1.84%, and 15.23 ± 3.32% (Figures 2(a) and 2(b)). To further investigate the mechanisms through which GLO1, NAC, or AG can protect HBMEC from MG plus OGD-induced cytotoxicity, we measured ROS production to detect their potential antioxidant effects. Interestingly, ROS level was found to be significantly increased in the MG plus OGD group as compared with the OGD group alone (24.75 ± 1.72% vs. 11.53 ± 1.15%) (Figures 2(c) and 2(d)), whereas ROS release was significantly decreased upon the pretreatment of GLO1, NAC, or AG (9.04 ± 2.36%, 10.75 ± 1.82%, and 9.89 ± 1.55%). The results clearly suggested that the antiapoptotic protective effects of GLO 1, NAC, or AG on HBMECs were partially because of significant attenuation in ROS production.

### 3.3. Impact of GLO1, NAC, or AG on HBMEC Migration

**Figure 3:**
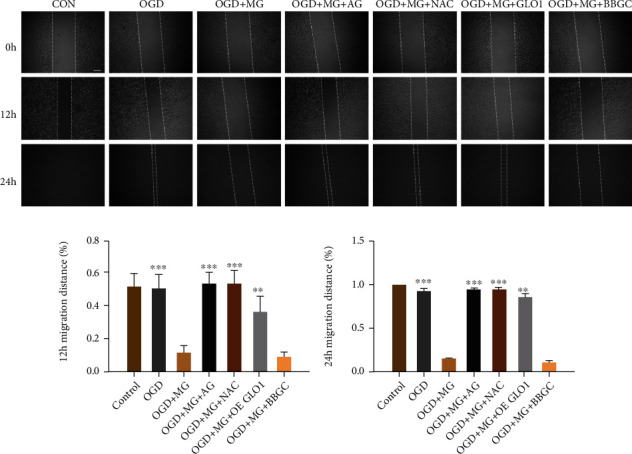
Impact of GLO1, NAC, or AG on HBMEC migration. (a) The representative images of HBMEC migration were monitored at 0, 12, and 24 hours after different treatment conditions. (b, c) The distance of cellular migration was calculated to quantitatively determine the migration ability of the cells. All data are the means ± SD (*n* = 3), ^∗^*p* < 0.05, ^∗∗^*p* < 0.01, and ^∗∗∗^*p* < 0.001 vs. the MG group. Scale bar = 5 mm.

Cell migration is one of the key aspects of endothelial cell biology and has been reported to be associated with angiogenesis. Given that HBMEC is pathologically relevant to ischemic stroke, function recovery and MG may contribute to endothelial dysfunction leading to an aggravated diabetic cerebral damage. A previous study has demonstrated that MG levels can significantly accumulate and impair neovascularization in ischaemic tissues during diabetes. We next investigated the impact of MG in HBMEC migration potential and whether GLO1, NAC, or AG has any effect on the cellular migration impaired by MG. As shown in Figure 3, OGD plus MG resulted in a significant reduction in the migration of HBMEC as compared with OGD-treated cells. In contrast, GLO1, NAC, or AG pretreatment significantly attenuated cell migration inhibition induced by MG plus OGD exposure.

### 3.4. Effects of GLO1, NAC, or AG on HBMEC Tube Formation

**Figure 4:**
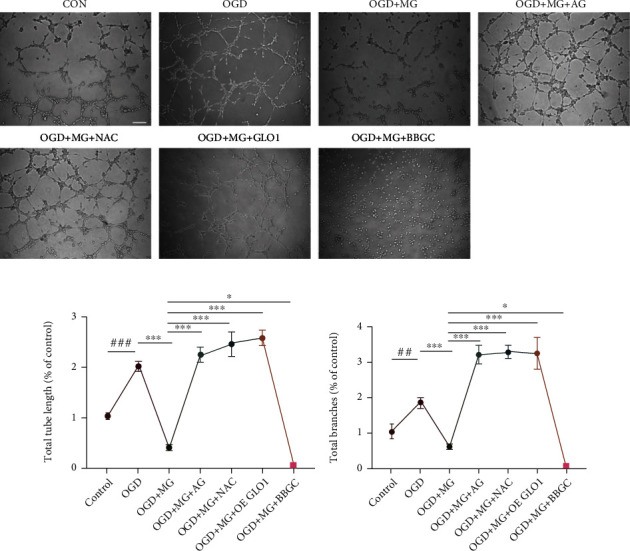
The potential effects of GLO1, NAC, or AG on HBMEC tube formation. (a) Representative images of HBMEC tube formation was monitored at 4 hours after different treatment conditions. (b, c) Quantitative analyses expressed as both total tube length and total number of branches. All data are the means ± SD (*n* = 3), ^∗^*p* < 0.05, ^∗∗^*p* < 0.01, and ^∗∗∗^*p* < 0.001 vs. the MG group. Scale bar = 5 mm.

In our study, we have clearly demonstrated the impairment in HBMEC migration caused by MG. To further evaluate the function of MG in the angiogenic process, a tube formation assay was performed in HBMEC under MG plus OGD stress conditions. As shown in Figure 4, MG plus OGD exposure significantly decreased the number of tubes formed in cultured HBMEC as compared with OGD-treated cells. GLO1, NAC, or AG pretreatment significantly increased the number of tubes formed in the cultured HBMEC as compared to that from the respective controls. These results indicated that MG-induced oxidative stress may impair the angiogenic function of HBMECs and GLO1, NAC, or AG can effectively reverse its dysfunction in the angiogenic process.

### 3.5. GLO1, NAC, or AG Protects against Decrease in the Levels of Angiogenesis Factors Induced by MG Treatment

**Figure 5:**
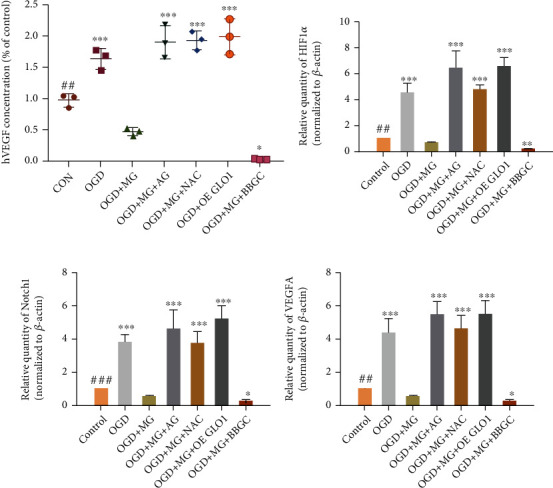
GLO1, NAC, or AG pretreatment can protect against MG-induced decrease in the mRNA levels of angiogenesis factors. (a) ELISA of secreted VEGF in HBMEC as indicated. (b–d) Bar charts showed quantitative PCR data about the relative HIF-1*α*, Notch1, and VEGFA levels. All data are the means ± SD (*n* = 3), ^∗^*p* < 0.05, ^∗∗^*p* < 0.01, and ^∗∗∗^*p* < 0.001 vs. the MG group.

**Figure 6:**
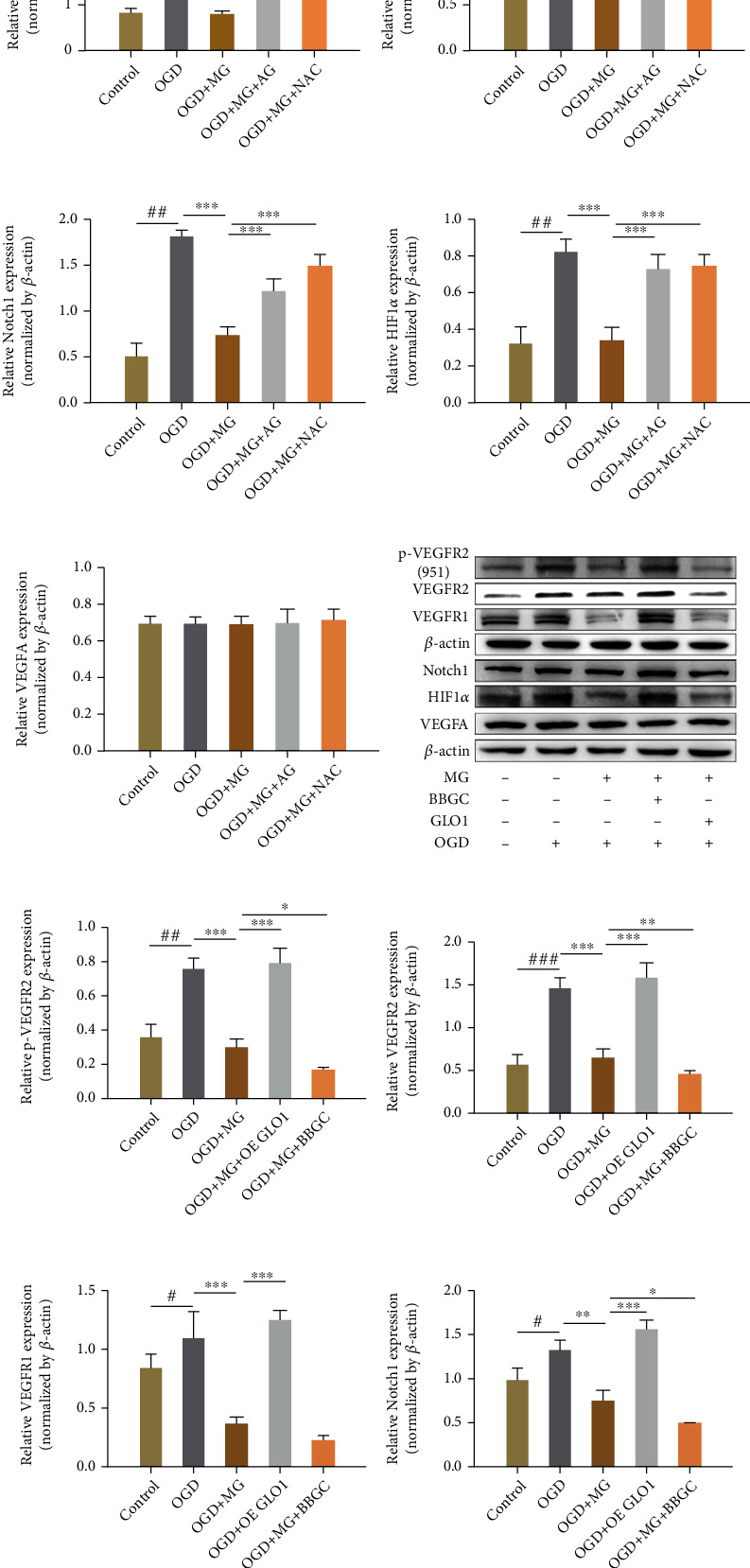
GLO1, NAC, or AG pretreatment can protect against MG-induced decrease in the protein levels of angiogenesis factors. (a, h) Western blot showed angiogenesis factors protein expression. (b–g, i–n) Quantitative analysis of p-VEGFR2-951, VEGFR2, VEGFR1, Notch1, HIF-1*α*, and VEGFA protein levels. All data are the means ± SD (*n* = 3), ^∗^*p* < 0.05, ^∗∗^*p* < 0.01, and ^∗∗∗^*p* < 0.001 vs. the MG group.

To understand the mechanism of impaired angiogenesis with MG, the activities of the various angiogenesis factors, namely, VEGFA, p-VEGFR2 (951), VEGFR2, VEGFR1, HIF-1*α*, and NOTCH1 were tested in qRT-PCR, ELISA, and western blotting. Interestingly, incubation of HBMEC with MG (1 mmol/L) plus OGD reduced VEGFA, HIF-1*α*, and Notch1 mRNA expression as noted in the qRT-PCR assay, which was significantly prevented by pretreatment with GLO I, NAC, or AG (Figures 5(b)–5(d)). At the same time, our data showed that p-VEGFR2(951), VEGFR2, VEGFR1, HIF-1*α*, and Notch1 protein expression was decreased in MG plus OGD-treated cells as compared with only OGD-treated cells, and this decrease could be effectively reversed by GLO1, NAC, or AG treatment (Figures 6(a)–6(n)). The result was also verified in ELISA, thereby indicating that the reduced human VEGF expression in HBMECs upon MG plus OGD exposure could lead to a substantial angiogenesis dysfunction (Figure 5(a)).

### 3.6. NAC or AG Protects against Inhibitory Effect of MG in Chorioallantoic Membrane (CAM) Assay

**Figure 7:**
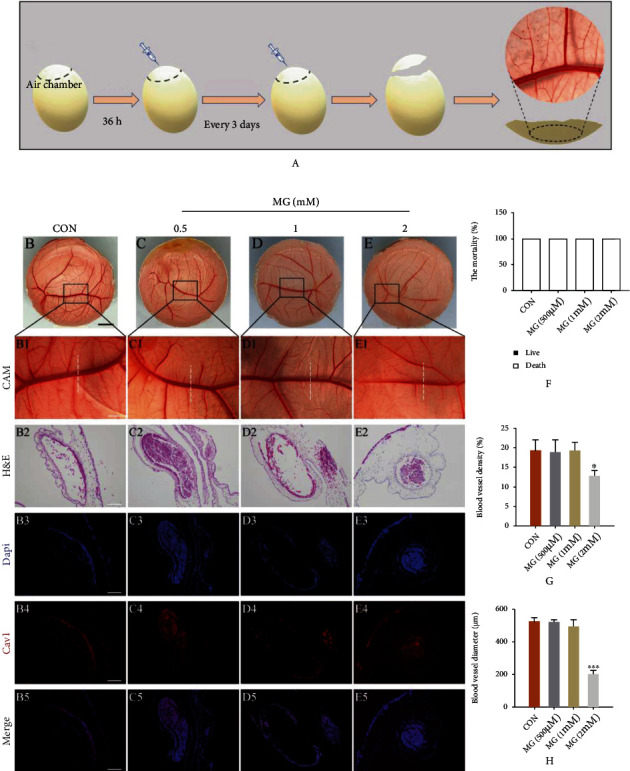
The chick embryo chorioallantoic membrane (CAM) assay was designed to study the antiangiogenesis capacity of MG. (a) A diagrammatic sketch depicting how and when to inject the drugs and how embryos were harvested from the eggs. (b–e, b1–e1) Representative bright-field images of chicken embryo CAM vascular plexus were taken from the control, 0.5 mmol/L MG, 1 mmol/L MG, and 2 mmol/L MG groups. (b2–e2) The representative H&E stained cross sections of the parts have been indicated by the lines in (b1)–(e1), respectively. DAPI (b3–e3), Cav1 (b4–e4), and merged (b5–e5) immunofluorescence staining shows the cross section of the part indicated by the lines in (b1)–(e1). (f) Statistical analysis of chicken embryo mortality. (g) The vascular plexus densities on chick embryo CAM. (h) Comparison of chick embryo CAM vessel diameter. All the data shown are the means ± SD (*n* = 3), ^∗^*p* < 0.05, ^∗∗^*p* < 0.01, and ^∗∗∗^*p* < 0.001 vs. the control group. Scale bars = 5 mm in (b)–(e); 2 mm in (b1)–(e1); 100 *μ*m in (b2)–(e5).

**Figure 8:**
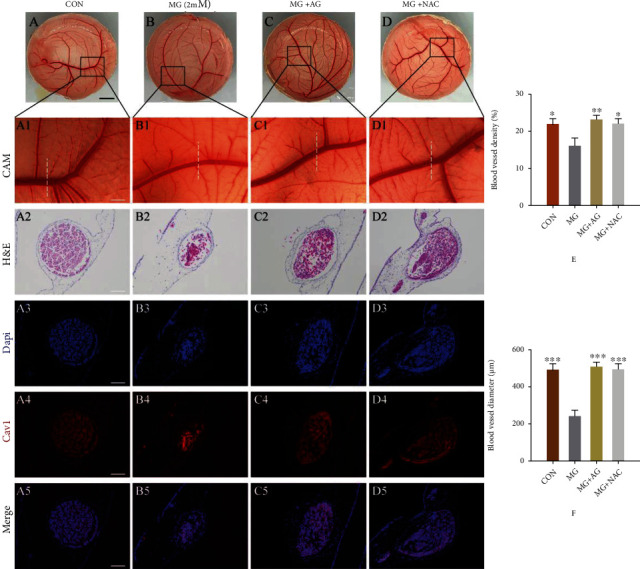
NAC or AG can protect against the inhibitory effect of MG observed in chorioallantoic membrane (CAM) assay. (a–d, a1–d1) Representative bright-field images of chicken embryo CAM vascular plexus were taken from the control, 2 mmol/L MG, MG+AG, and MG+NAC groups. (a2–d2) The representative H&E-stained cross sections of the parts indicated by the lines in (a1)–(d1), respectively. DAPI (a3–d3), Cav1 (a4–d4), and merged (a5–d5) immunofluorescence staining shows the cross section of the part indicated by the line in (a1)–(d1). (e) The density of vascular plexus on chicken embryo CAM. (f) A comparison of chick embryo CAM vessel diameter. All data are the means ± SD (*n* = 3), ^∗^*p* < 0.05, ^∗∗^*p* < 0.01, and ^∗∗∗^*p* < 0.001 vs. the MG group. Scale bars = 5 mm in (a)–(d); 2 mm in (a1)–(d1); 100 *μ*m in (a2)–(d5).

The chick embryo chorioallantoic membrane (CAM) is an extraembryonic membrane that is an ideal model for in vivo research related to angiogenesis. In our study, CAM assay was designed to study the antiangiogenesis potential of MG. The process of new blood vessel formation and possible response upon exposure to antiangiogenic compound MG was determined. As shown in Figures 7(c) and 7(d), the density formation of vascular plexus was not significantly different between the MG- (0.5, 1 mmol/L) treated group and the control group. However, the concentration of 2 mmol/L MG showed a significant reduction of density formation of vascular plexus in the MG-treated group as compared with the control group. Similarly, we observed that the new blood vessel diameter was significantly smaller in the MG-treated group as compared with the control group by using IF and HE staining (Figures 7(b2)–7(e5)). Interestingly, when the CAM was pretreated with NAC (2 mmol/L) or AG (2 mmol/L), the quantity of vessel plexus was observed to be increased by 9.02% and 10.40%, respectively (Figure 8(e)). Interestingly, the new blood vessel diameter showed a significant induction in NAC- or AG-treated group as compared with the control group (Figure 8(f)). These results displayed that MG can substantially inhibit angiogenesis process in vivo and NAC or AG may exhibit a protective effect against angiogenesis dysfunction induced by MG.

### 3.7. Effects of NAC or AG on mRNA and Protein of AGEs/RAGE and Angiogenesis Factor Expression

**Figure 9:**
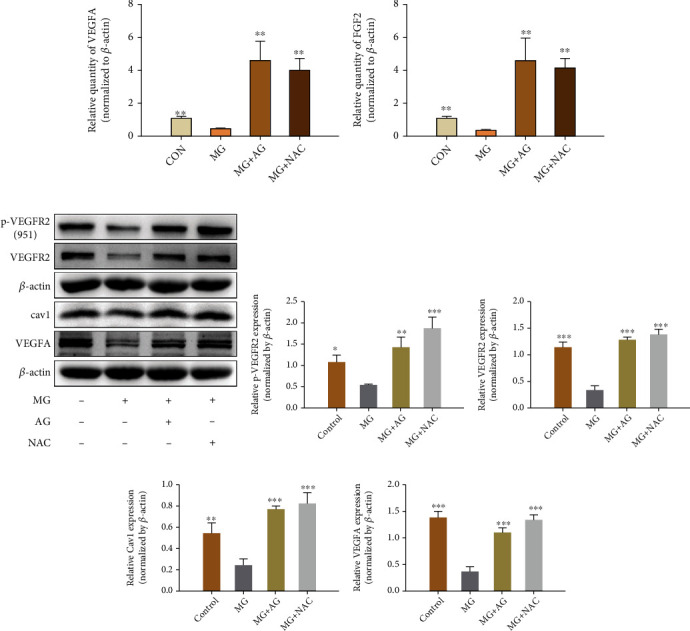
Effects of NAC or AG on the expression of AGEs/RAGE and angiogenesis factors at mRNA and protein levels. (a, b) Bar charts showing quantitative RT-PCR data about the relative levels of VEGFA (a) and FGF2 (b) expressions (normalized to *β*-actin). (c) Western blotting showing AGEs/RAGE and angiogenesis-related protein expression. (d–g) Quantitative analysis of p-VEGFR2-951 (d), VEGFR2 (e), Cav1 (f), VEGFA (g), and protein expression levels. All data are the means ± SD (*n* = 3), ^∗^*p* < 0.05, ^∗∗^*p* < 0.01, and ^∗∗∗^*p* < 0.001 vs. the MG group.

qRT-PCR and western blotting analysis was utilized to evaluate the effects of NAC or AG on VEGFA and FGF-2 expression after MG induction in the CAM model. As shown in Figures 9(a) and 9(b), treatment with MG (2 mmol/L) led to a significant reduction in VEGFA and FGF2 mRNA expression as compared with the vehicle control group. We also observed that the protein level of VEGFA, p-VEGFR2 (951), VEGFR2, and caveolin-1 decreased in the MG-treated group as compared to the vehicle control group. Pretreatment with NAC and AG in the MG-treated group led to a significant increase in VEGFA, FGF2 mRNA and VEGFA, p-VEGFR2(951), VEGFR2, and caveolin-1 protein expression compared with that in the MG group. The changes in protein expression of AGEs/RAGE were found to be just the opposite.

## 4. Discussion

**Figure 10:**
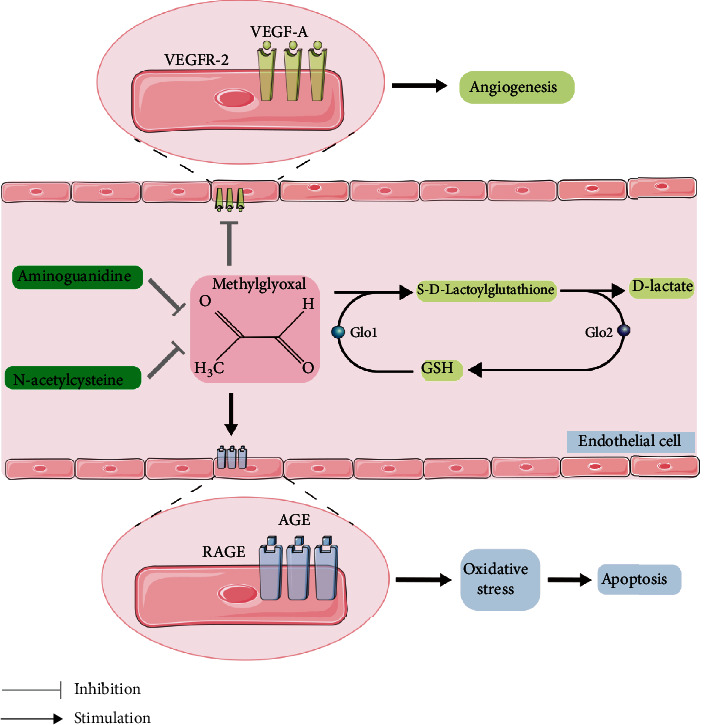
The proposed mechanism illustrating how NAC, AG, and GLO1 could significantly improve the angiogenesis dysfunction induced by MG exposure.

In this study, we have reported that MG, a glucose metabolite, can induce cellular dysfunctions and angiogenesis in OGD cultured HBMEC as well as in CAM. The effects of MG on cultured HBMEC and CAM were attenuated by GLO 1 treatment and exposure to two different MG scavengers, AG and NAC. Thus, our results provide a substantial evidence linking high level of MG to angiogenic dysfunction. Exposure to MG reduced cell viability, cell migration, and tube formation, as well as promoted cellular apoptosis in cultured HBMEC under OGD. The possible mechanism can be attributed to interaction of MG with AGEs/RAGE thereby leading to an increase in oxidative stress in HBMEC and CAM. We further observed that MG treatment resulted in significant reduction of the density of blood vessel in CAM. Treatment with GLO 1, NAC, and/or AG significantly enhanced angiogenesis, as well as restored impaired endothelial cell function. Moreover, we have also elegantly demonstrated that GLO 1, NAC, or AG can promote angiogenic response after MG treatment and also upregulated the expression of angiogenic factor HIF-1a, VEGF, VEGFR-2, Notch1, and p-VEGFR-2 (Figure 10).

Increasing studies have shown that diabetes can increase the risk and worsen the outcome of stroke [22, 23]. Diabetes is associated with large and small vessel damage within the brain. In recent years, few studies have reported that MG may act as an important factor leading to diabetes micro-/macrovascular dysfunction and cardiovascular disease. Vascular dysfunction induced by diabetes is most likely to be mediated by MG. For example, it has been found that the exposure to increased MG in vivo may be associated with the onset of microvascular damage and other diabetes-like complications in the absence of hyperglycemic context [24]. Wang et al. showed that diabetic mice showed aggravation in poststroke brain injury and this injury could be attenuated by MG elimination via the GSH-dependent mechanism. In addition [25], the positive correlation between higher plasma MG levels and higher incidences of cardiovascular disease supports a key role for MG in stroke exacerbation in diabetes [26]. Thus, our findings suggest that MG may be a critical determinant of stroke outcome in diabetes.

To the best of our knowledge, the effect of MG on angiogenesis under ischemic stroke has not been reported previously. In the present study, we investigated if MG treatment could induce damage on the microvessels present in the brain and angiogenesis dysfunction in vivo. We have provided conclusive evidence that MG alone exerted a dose-dependent damage in cultured HBMEC, which was consistent with previously reported findings [27]. AGEs have been found to be related to the progression of vascular disease in diabetic patients [28]. Previous studies have shown that MG-induced vascular endothelial cell damage can be caused by an increased expression of AGEs and its receptor RAGE [29]. RAGE antagonism studies have revealed that it may be beneficial for protective effects and management of diabetes-induced vascular complications [30]. Our results demonstrated that AGE/RAGE accumulation was significantly increased 24 h after MG treatment. Intriguingly, GLO 1, NAC, or AG which are known to impair cellular functions by scavenging ROS could significantly decrease AGE/RAGE accumulation. We have also established that MG promoted the cytotoxicity under OGD condition and significantly increased the expression of AGEs and RAGE, which was protected by exposure to GLO 1, NAC, or AG. Recent studies have revealed that hyperglycemia can significantly inhibit peripheral limb ischemia repair and the neovascularization process [31]. Indeed, it has been demonstrated that AGE/RAGE restriction may improve angiogenesis and wound healing in endothelial cells and diabetic animal model [32, 33]. In particular, the relevance of MG exposure can lead to an impairment of wound healing and angiogenesis [34]. Moreover, it has been demonstrated that the overexpression of GLO1 can reduce AGE formation in cultured endothelial cells [35] and also improve hyperglycemia-induced impairment of angiogenesis in human microvascular ECs [36].

However, in the current study, for the first time, we have used an exogenous exposure of MG on the HBMEC and CAM to demonstrate the impairment of MG overload on angiogenesis in vitro and in vivo, which was significantly restored by GLO 1, NAC, or AG treatment. During angiogenesis, angiogenic factors play an essential role in the development of neovascularization process. For instance, abrupt changes in VEGF and VEGFR1 levels have been shown to contribute to the impaired angiogenesis after ischemia in a type 2 diabetes mellitus mouse model [37]. We also observed a significant decrease in the VEGF, VEGFR1, and VEGFR2 levels after MG plus OGD treatment compared with OGD treatment. In addition, it was reported that HIF-1*α* interaction with VEGF-Notch1 can also play a very important role in regulating neurovascular regeneration after cerebral ischemia [38, 39]. However, we noted that MG treatment in HBMEC significantly reduced the protein expression of HIF-1*α*, VEGF, and Notch1, which was prevented by GLO 1, NAC, or AG treatment in HBMEC. This was consistent with previous studies indicating that MG can impair the expression of angiogenic factors. These results suggested that MG may function as a significant factor leading to impairment of angiogenesis in diabetes.

Chick chorioallantoic membrane (CAM) assays have been extensively used to investigate the influence of chemicals/compounds or detrimental factors on the process of angiogenesis [21]. Thus, we used an embryonic angiogenesis model to study whether NAC or AG can reduce the observed effect of MG on angiogenesis. The effect of MG administration on angiogenesis revealed that a lower concentration (500 *μ*mol/L, 1 mmol/L) of MG did not impair angiogenesis but at the higher concentrations (2 mmol/L) could significantly attenuate the angiogenesis. Angiogenesis-related genes, including *VEGFA*, *VEGFR2*, *p-951-VEGFR2*, and *caveolin 1*, were also found to be reduced after MG exposure to CAM. In addition, AGEs and RAGE protein expression was also increased upon MG treatment, which has been reported to contribute to oxidative stress and angiogenesis dysfunction [40, 41]. A previous study has demonstrated that NAC as a potential antioxidant can significantly reverse pathological angiogenesis by causing modulation of oxidative stress in the CAM model [42]. In our study, we found that NAC or AG was able to effectively prevent angiogenesis dysfunction after MG treatment in the CAM model, which was partially due to inhibition of oxidative stress induced by interaction of MG with AGE/RAGE.

## 5. Conclusion

In conclusion, our study clearly established that exposure to MG had a significant impact on angiogenesis when the cells were combined with ischemia and hypoxia injury. HBMECs were cocultured with MG, which led to an increased accumulation of AGEs as well as augmented expression of RAGE. The inhibition of angiogenesis in the MG plus OGD group may have contributed to an increased interaction of MG with AGEs/RAGE thereby promoting enhanced ROS release. These changes were found to be partially prevented by pretreatment with GLO 1, NAC, or AG. Therefore, these findings may form the basis of novel treatment options for diabetes-related complications due to the presence of ischemic stroke.

## Data Availability

The data used to support the findings of this study are included within the article.

## Supplementary Materials

Supplementary Materials Figure S1: detection of infection capability of overexpression GLO1 virus. A1-A2: after 72 hours of the viral infection (MOI = 100), image of the control cell under the white light and fluorescence. Figure B1-B2: the image of the cell infected with the overexpressing GLO1 viral group. Figure C: the PCR results of GLO1 expression abundance of the two different groups of the cells (*p* < 0.001). Scale bars = 200 *μ*m in A1-B2.
