# Supplementary material for: Methylglyoxal Scavengers Attenuate Angiogenesis Dysfunction Induced by Methylglyoxal and Oxygen-Glucose Deprivation
Source: Oxid Med Cell Longev. 2022 Jan 5;2022:8854457. doi: 10.1155/2022/8854457 (PMC8754597; doi:10.1155/2022/8854457)
Supplement: Supplementary Materials — Figure S1: detection of infection capability of overexpression GLO1 virus. A1-A2: after 72 hours of the viral infection (MOI = 100), image of the control cell under the white light and fluorescence. Figure B1-B2: the image of the cell infected with the overexpressing GLO1 viral group. Figure C: the PCR results of GLO1 expression abundance of the two different groups of the cells (p < 0.001). Scale bars = 200 μm in A1-B2. [file 8854457.f1.docx]

**Supplementary Information**

**Methylglyoxal scavengers attenuate angiogenesis dysfunction induced by methylglyoxal and oxygen-glucose deprivation**

Authors: Wei Chen ^1,2^, Wenhui Huang ^1,2^, Yang Yu^1,2^, Keshen Li ^1,2,*^

^1^ Clinical Neuroscience Institute of Jinan University, Guangzhou, 510632,China;

^2^Department of Neurology and Stroke Center, The First Affiliated Hospital, Jinan University, Guangzhou ,510632, China

*Correspondence: likeshen1971@126.com

**Supplementary Results**

*1: Detection of infection capability of overexpression GLO1 virus*

The viral infection was detected by fluorescence expression abundance under a fluorescence microscope. As shown in Figure S1, the cells were infected with the virus under the condition of MOI=100. After 72 hours of infection, the fluorescence expression abundance reached approximately 90%. Real-time PCR experiment was thereafter used to verify the efficiency of the viral infection, and the expression of GLO1 was calculated by 2^-ΔΔCt^. As shown in Figure S1(C), the expression abundance of the overexpression GLO1 viral group was 106.419 times that of the control group (p<0.001), and the expression was found to increase significantly.


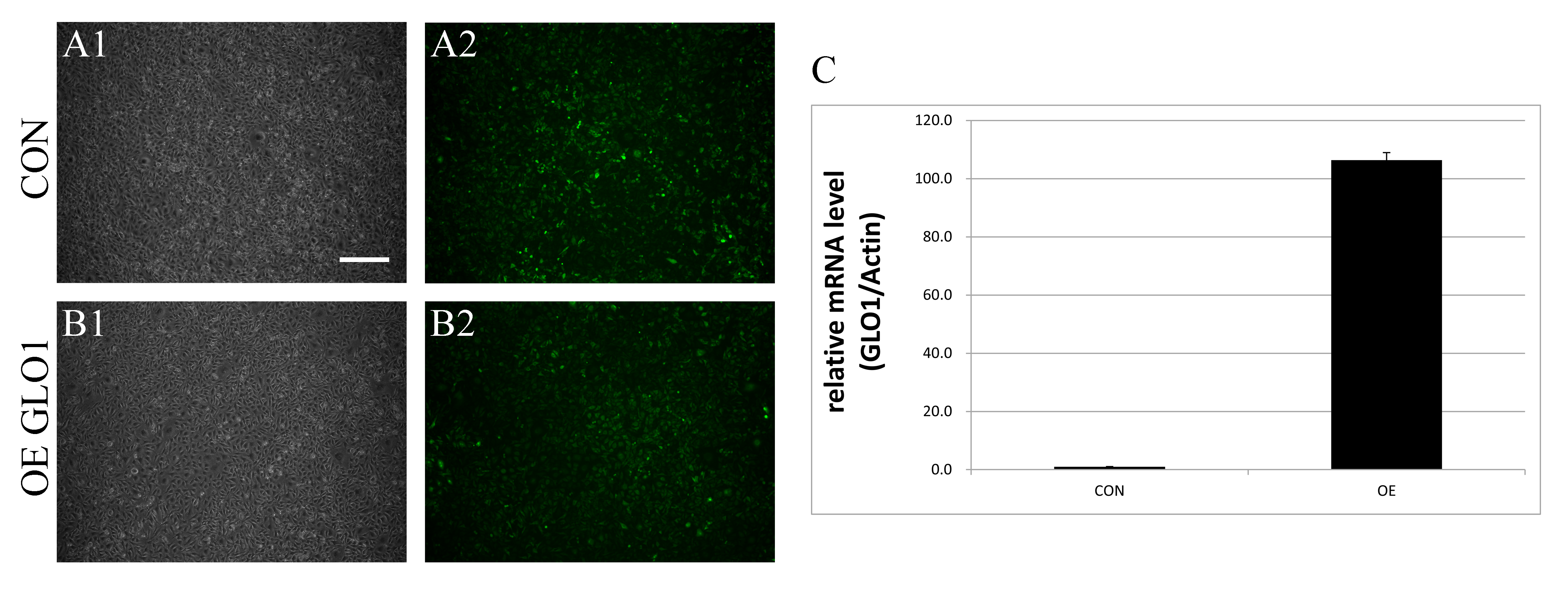


**Figure S1.** Detection of infection capability of overexpression GLO1 virus. A1-A2: After 72 hours of the viral infection (MOI=100), image of the control cell under the white light and fluorescence. Figure B1-B2: The image of the cell infected with the over-expressing GLO1 viral group. Figure C: The PCR results of GLO1 expression abundance of the two different groups of the cells (p＜0.001). Scale bars = 200 μm in A1-B2.
